# Multiple epiphyseal dysplasia mutations in *MATN3* cause misfolding of the A-domain and prevent secretion of mutant matrilin-3

**DOI:** 10.1002/humu.20263

**Published:** 2005-12

**Authors:** Sally L Cotterill, Gail C Jackson, Matthew P Leighton, Raimund Wagener, Outi Mäkitie, William G Cole, Michael D Briggs

**Affiliations:** 1 Wellcome Trust Centre for Cell-Matrix Research, Faculty of Life Sciences, University of ManchesterManchester, United Kingdom; 2 Center for Biochemistry, University of CologneCologne, Germany; 3 Hospital for Children and Adolescents, University of HelsinkiHelsinki, Finland; 4 Hospital for Sick ChildrenToronto, Ontario, Canada

**Keywords:** cartilage, chondrodysplasias, matrilin-3, gene mutation, protein misfolding, chaperone protein, disease mechanism, multiple epiphyseal dysplasia

## Abstract

Multiple epiphyseal dysplasia (MED) is a relatively common skeletal dysplasia that can present in childhood with a variable phenotype of short stature and pain and stiffness in the large joints, and often progresses to early-onset osteoarthritis in adulthood. Mutations in the matrilin-3 gene (*MATN3*) have recently been shown to underlie some forms of autosomal dominant MED. To date all MED mutations in matrilin-3 cluster in the single A-domain, suggesting that they may disrupt the structure and/or function of this important domain. To determine the effects of *MATN3* mutations on the structure and function of matrilin-3 we expressed both normal and mutant matrilin-3 in mammalian cells. Wild-type (wt) matrilin-3 was efficiently secreted into conditioned medium, whereas mutant matrilin-3 was retained and accumulated within the cell. Furthermore, when the mutant A-domains were examined individually, they existed primarily in an unfolded conformation. Co-immunoprecipitation experiments demonstrated that the mutant A-domains were specifically associated with ERp72, a chaperone protein known to be involved in mediating disulfide bond formation. Light microscopy of cartilage from an MED patient with a *MATN3* mutation showed the presence of intracellular material within the chondrocytes, whilst the overall matrix appeared normal. On electron micrographs, the inclusions noted at the light microscopy level appeared to be dilated cisternae of rough endoplasmic reticulum and immunohistochemical analysis confirmed that the retained protein was matrilin-3. In summary, the data presented in this paper suggest that MED caused by *MATN3* mutations is the result of an intracellular retention of the mutant protein.

## INTRODUCTION

Extracellular matrix (ECM) proteins typically comprise several different structural domains that often occur in multiples [Hohenester and Engel, 2002]. In some instances the same domain can be found in many different proteins, each of which has a distinct function. One such protein domain is the von Willebrand factor A-domain (vWFA) [Whittaker and Hynes, 2002]. This domain was originally identified in von Willebrand factor, a soluble plasma protein that plays a role in blood clotting by mediating platelet adhesion to the collagen fibrils of damaged blood vessels. Von Willebrand factor A-domains (A-domains) are also found in collagen types VI, type VII, type XII, type XIV, type XVII, type XX, type XXI, and other ECM proteins such as matrilins 1–4, cochlin, vitrin, and WARP [Fitzgerald et al., 2002; Wagener et al., 2005; Whittaker and Hynes, 2002]. A homologue of the A-domain is the integrin inserted-domain or I-domain, which is found in many α and all β integrins [Dickeson and Santoro, 1998].

The A-domain consists of approximately 200 amino acids arranged in a Rossman or dinucleotide-binding fold, with five parallel β-strands and one antiparallel β-strand at the center of the domain surrounded by seven α-helices on the external faces of the domain [Whittaker and Hynes, 2002]. Many A-domains can coordinate a metal ion using noncontiguous residues at the top of the domain called the metal ion-dependent adhesion site (MIDAS) motif.

A number of human diseases are caused by the mutation of residues within the A-domains of various proteins. A missense mutation (p.Gly1679Glu) in the A-domain of the α3 chain of type VI collagen was found in patients with Bethlem myopathy (MIM# 158810) [Pan et al., 1998] and has been shown to result in reduced secretion and increased degradation of mutant a3(VI) chains [Sasaki et al., 2000]. Furthermore, a missense mutation in the α3 domain of von Willebrand factor results in reduced collagen binding by the mutant protein and an inherited hemorrhagic syndrome, while the mutant monomers were still able to assemble into multimers and bind glycoprotein 1b [Ribba et al., 2001]. Overall, these data suggest that the type and location of a particular mutation within the A-domain determine the specific disease mechanism and ultimately the phenotypic consequence.

Multiple epiphyseal dysplasia (MED) is a skeletal dysplasia of varying severity and is characterized by stiffness and pain in the large joints, which often results in early-onset osteoarthritis [Briggs and Chapman, 2002; Unger and Hecht, 2001]. Several genes have been implicated in autosomal dominant MED, including those encoding cartilage oligomeric matrix protein (COMP; MIM# 132400) [Briggs et al., 1995], all three α-chains of type IX collagen (*COL9A1, COL9A2*, and *COL9A3*; MIM# 120210, 600204, and 600969, respectively) [Czarny-Ratajczak et al., 2001; Muragaki et al., 1996; Paassilta et al., 1999] and matrilin-3 (*MATN3*; MIM# 607078) [Chapman et al., 2001].

Matrilin-3 is a modular protein found specifically in the ECM of cartilage [Wagener et al., 1997]. It comprises a single A-domain, four EGF repeats, and a coiled-coil domain that facilitates oligomerization. It is the third member of the matrilin family of proteins, and forms either homotetramers or heterotetramers with matrilin-1 [Wu and Eyre, 1998; Klatt et al., 2000]. Matrilin-3/1 isolated from cartilage has been shown to bind with high affinity to both COMP and type IX collagen [Briggs and Chapman, 2002; Mann et al., 2004].

The gene encoding matrilin-3 (*MATN3*) is located on chromosome 2 and comprises eight exons [Belluoccio et al., 1998]. Several missense mutations in *MATN3* have been associated with MED, including p.Arg121Trp, p.Val194Asp [Chapman et al., 2001], p.Thr120Met, p.Glu134Lys, p.Ile192Asn, and p.Ala219Asp [Jackson et al., 2004]. In addition, we also identified a nonsynonymous polymorphism, p.Glu252Lys [Jackson et al., 2004], which apparently does not cause MED but may play a role in modifying disease severity in some patients. All of these missense mutations and the polymorphism are found in the single A-domain of matrilin-3, which is encoded by exon 2 of *MATN3*. Interestingly, a form of hand osteoarthritis (MIM# 607850) has been linked to a p.Thr303Met mutation located in the first EGF repeat of matrilin-3 [Stefansson et al., 2003], while a recessive form of spondyloepimetaphyseal dysplasia (MIM# 608728) results from homozygosity for a mutation (p.Cys304Ser) at the neighboring residue [Borochowitz et al., 2004].

To understand the effect of MED-causing mutations on the structure and function of matrilin-3 and thereby gain insight into the disease mechanisms of this form of MED, we expressed both normal and mutant full-length matrilin-3 proteins and single A-domains in mammalian cells. This approach allowed us to investigate the effects of mutations on the folding and trafficking of matrilin-3 and propose a potential disease mechanism for this form of MED.

## MATERIALS AND METHODS

### Cloning of Normal and Mutant Matrilin-3 A-Domains

PCR amplification of exon 2 of *MATN3*, which encoded the entire A-domain, was performed using primers MATN3ex2F (5′-ggcccagccggcctgcaagagcagacccttggac-3′, *Sfi* I restriction site underlined) and MATN3ex2R (5′-gcggccgcacagaaggtttcctggaatct-3′, *Not* I restriction site underlined) using a standard protocol. Genomic DNA from MED patients with *MATN3* mutations (p.Arg121Trp, p.Val194Asp, p.Thr120Met, p.Glu134Lys, p.Ile192Asn, and p.Ala219Asp) and the nonsynonymous polymorphism (p.Glu252Lys) was used as the template in the PCR. In brief, PCR amplifications were performed in 25 μl reactions containing 0.5 μM of each primer, 0.625 U of *Taq* DNA polymerase, 67 mM Tris-HCl (pH 8.0 at 25°C), 16 mM (NH_4_)_2_SO_4_, 3.7 mM MgCl_2_, 0.085 mg/ml BSA, 6.7 μM EDTA, 0.75 mM of each dNTP, and 50–100 ng of genomic DNA. PCR cycling consisted of initial denaturation at 95°C for 3 min followed by 35 cycles of 95°C for 1 min, 62°C for 1 min, and 72°C for 1 min and a final extension at 72°C for 5 min. PCR amplified DNA fragments were purified using the QIAquick® PCR purification kit (Qiagen Ltd., Crawley, West Sussex; http://www1.qiagen.com) and individual alleles were cloned using the TA cloning kit (Invitrogen Ltd., Paisley, UK; http://www.invitrogen.com) following the manufacturer's instructions. Clones containing only the nucleotide change of interest, as identified by bi-directional DNA sequencing, underwent double restriction digest with *Sfi* I and *Not* I and were sub-cloned into pSecTag2A (Invitrogen).

### Cloning of Normal and Mutant Full-Length Matrilin-3

Human full-length matrilin-3 cDNA (a kind gift from D. Krakow) was digested with P*st* I and subcloned into the pCMVTAG4 (Stratagene, La Jolla, CA; http://www.stratagene.com) vector (FLM3-CMV). PCR amplification to remove the stop codon was then performed using M3XbaF (5′-ctttcctctagattccaggaa-3′, *Xba* I restriction site underlined) and M3R (5′-aagcttacgatgtatttgtccat attc-3′, *Hind* III restriction site underlined) using a standard protocol with P*fu* DNA polymerase. The PCR amplified DNA fragment was purified using the QIAquick® PCR purification kit (Qiagen) and then double digested with *Xba* I and *Hind* III. The digested fragment replaced the original *Xba* I/*Hind* III fragment in FLM3-CMV to create WT-FLM3 (wt full-length matrilin-3) with no stop codon and in-frame with the FLAG tag. p.Val194Asp and p.Glu252Lys mutations were introduced by replacing a *Eco* NI/*Xba* I fragment from WT-FLM3 with *Eco* NI/*Xba* I fragments from the respective mutant A-domains (see previous) using standard protocols. All clones were sequenced to confirm that they contained only the desired mutation.

### Cell Culture and Transfection

CHO-B2 cells were cultured in Ham's F12 media supplemented with 10% FBS, 2mM *L*-glutamine, 100 U/ml penicillin, and 100 μg/ml streptomycin (Biowhittaker; http://www.bioresearchonline.com). The cells were incubated at 37°C in humidified air containing 5% CO_2_.

The cells were transfected using Lipofectin (Invitrogen) according to the manufacturer's instructions. Briefly, when the cells were 50–70% confluent monolayers in 75 cm^2^ flasks they were incubated with 2 μg of construct DNA and Lipofectin in Opti-MEM serum-free media (Invitrogen) for 24 hr. To obtain stable cell lines the cells were transferred to media containing 250 μg/ml Zeocin (Invitrogen) for selection. This media was changed every 2–3 days until distinct foci of individual clones resistant to Zeocin could be seen (after ∼2 weeks). Individual clones were removed by incubation with trypsin, expanded in 6-well plates and when confluent transferred into 25 cm^2^ flasks. Conditioned media and cell lysates were tested for expression of the A-domain and individual cell lines that expressed the A-domain to the highest extent were then used in subsequent experiments.

### SDS-PAGE and Western Blot Analysis

Cells were lysed by incubation in 500 μl RIPA lysis buffer (10 mM Tris, pH 7.4, 150 mM NaCl, 1% sodium deoxycholate, 0.1% SDS) on ice for 15 min. The cell monolayer was removed, centrifuged at 13,600 × g for 5 min and the supernatant was removed for analysis. Conditioned media and cell lysates were separated by SDS-PAGE on 4–12% Tris-Bis gels (Invitrogen). Proteins were transferred to nitrocellulose membranes and the recombinant A–domain was detected using an anti-c-myc monoclonal antibody, clone 9E10 (Roche Diagnostics Ltd. Lewes, East Sussex, UK; http://www.roche-applied-science.com) with antimouse HRP conjugate (Sigma-Aldrich Company Ltd., Gillingham, Dorset, UK; http://www.sigmaaldrich.com) as a secondary antibody. Protein bands were visualised with SuperSignal® West Dura extended duration substrate (Perbio; http://www.perbio.com).

### Co-immunoprecipitation With Chaperone Antibodies

Co-immunoprecipitation was carried out on cell lysates produced as above from 5 × 10^5^ cells transfected with the different A-domain constructs. Proteins were precipitated using antibodies against chaperones found in the rough endoplasmic reticulum (rER): PDI, ERp72 (Stressgen, San Diego, CA; http://www.stressgen.com/), Grp78, Grp94 (Santa Cruz Biotechnology, Santa Cruz, CA; http://www.scbt.com). Prior to immunoprecipitation, the cells were cross linked with dithiobis[succinimidyl propionate] (DSP) to stabilize protein complexes. Aliquots of cells were washed twice in PBS then resuspended in 250 μl PBS. DSP was added to 1 mM from a fresh stock made up in dry DMSO and incubated for 30 min at room temperature. To stop the cross-linking reaction, 10 μl of 1 M Tris-HCl, pH 7.4 was added and cells were incubated for a further 15 min. Finally, cells were washed twice in PBS and lysed with RIPA lysis buffer as described above ready for immunoprecipitation.

Lysates were diluted 10-fold with immunoprecipitation buffer (50mM Tris-HCl, pH 7.4, 1% Triton X-100, 150 mM NaCl, 2 mM EDTA). First, cell lysates were precleared by incubation with 0.25 μg rabbit IgG (goat IgG with anti-Grp78 and -Grp94) and 20 μl Protein A-agarose (Protein G PLUS-agarose with anti-Grp78 and -Grp94) (Santa Cruz Biotechnology) under rotation for 1 hr at 4°C. The mixture was centrifuged at 3,000 × g for 5 min to remove the beads and nonspecifically bound proteins. The appropriate chaperone antibody was added to the supernatant and incubated under rotation for 1 hr at 4°C. Then 20 μl of Protein A-agarose was added and the tubes were rotated at 4°C overnight. After overnight incubation, the tubes were centrifuged at 3,000 × g for 3 min and the supernatant was discarded. The pellet was washed and centrifuged three times in immunoprecipitation buffer. The final pellet was resuspended in 1 × SDS-PAGE loading buffer containing 100 mM dithiothreitol (DTT), heated at 100°C for 5 min and centrifuged at 6,000 × g for 30 sec. The supernatants were analyzed by Western blotting as described previously.

### Immunohistochemistry

An iliac crest biopsy from a patient with the p.Arg121Trp MED-causing mutation was stained using an anti-matrilin-3 antibody. Frozen sections (5–10 μm thick) were cut from the biopsy and mounted on superfrost slides. A Vectastain ABC (Rabbit IgG) kit (Vector Laboratories Ltd., Peterborough, UK; http://www.vectorlabs.com) was used according to manufacturer's instructions. Antibody staining was detected with a DAB peroxidase substrate kit (Vector Laboratories Ltd.) and counterstained with hemotoxylin.

## RESULTS

### MED Mutations Cluster in the A-Domain of Matrilin-3

Missense mutations in the gene encoding matrilin-3 cause an autosomal dominant form of MED and in total eleven different mutations have now been identified in 26 unrelated families with various forms of MED (see Table 1). All but one of these mutations affect residues comprising the β-strands of the A-domain; indeed, disease-causing mutations have now been identified in four of the six β-strands (βB, βC, βD, and βE) that comprise the single β-sheet. The most common mutation is p.Arg121Trp, which has been identified in approximately onethird of all MED patients with a *MATN3* mutation (9/26). Sufficient studies have now been performed to suggest that MED mutations in *MATN3* are predominantly located in exon 2, which encodes the single A-domain.

**TABLE I:** Mutations Identified in *MATN3* in 26 Families with MED*

			Number of families inwhich thismutation has been identified		
Nucleotide change	Predicted amino acid change	Location within the A-domain	Present study	Previous studies	Reference
c.314T>C	p.Phe105Ser	α1		1	Mabuchi et al. [2004]
c.359C>T	p.Thr120Met	β2		4	Jackson et al. [2004]; Mabuchi et al. [2004]
c.361C>T	p.Arg121Trp	β2	2	7	Chapman et al. [2001]; Jackson et al. [2004]; Mabuchi et al. [2004]; this study
c.368C>A	p.Ala123Lys	βB		1	Mabuchi et al. [2004]
c.382G>C	p.Ala128Pro	βB		1	Mostert et al. [2003]
c.400G>A	p.Glu134Lys	βC		1	Jackson et al. [2004]
c.575T>A	p.Ile192Asn	βD		1	Jackson et al. [2004]
c.581A>T	p.Val194Asp	βD		1	Chapman et al. [2001]
c.584C>A	p.Thr195Lys	βD	1		This study
c.652T>A	p.Tyr218Asn	βD	1		This study
c.656C>A	p.Ala219Asp	βE		1	Jackson et al. [2004]
c.754G>A	p.Glu252Lys^a^	α7	1	3	Jackson et al. [2004]; this study

* Matrilin-3 cDNA accession number in GenBank is NM_002381.3 and nucleotide numbering is from the start of translation (i.e., +1 corresponds to the A of the ATG translation initiation codon in the reference sequence). Nucleotide mutations are based on the cDNA sequence of *MATN3* and are preceded with a “c.” symbol before the number.

a A non-disease-causing polymorphism identified in unaffected family members and unrelated controls.

### Expression of Normal and Mutant Matrilin-3 Proteins

**Figure 1:**
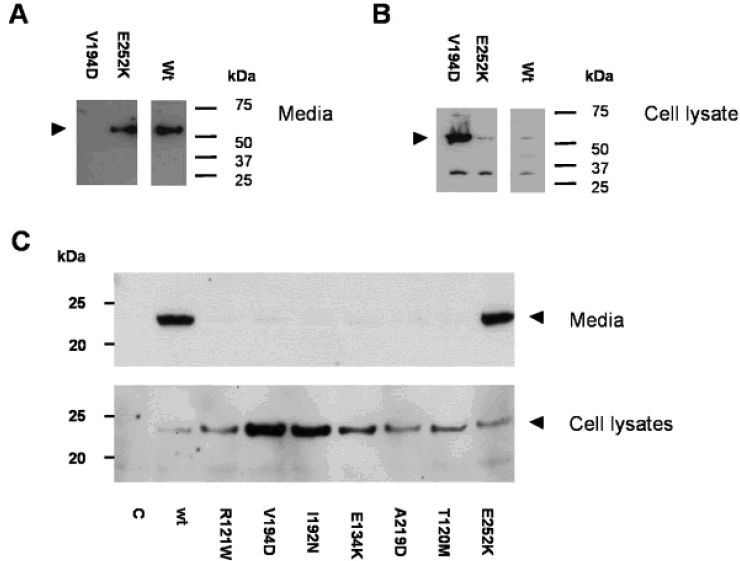
SDS-PAGE and Western blot analysis of conditioned media and cell lysates from CHO cells stably transfected with full-length matrilin-3 (**A** and **B**) and single A-domain (**C**) constructs. Gels were run under reducing conditions, and molecular weights are shown in kDa. A and B: Media and cell lysate samples from cells expressing full-length wt, a MED mutation (pVal194Asp), and the polymorphism (pGlu252Lys) are shown. Proteins were detected with an anti-FLAG antibody, and the full-length protein is indicated with an arrowhead.C:Untransfected cells (C) and cells transfected with normal (wt) and polymorphic (pGlu252Lys) A-domain are shown.The A-domain missense mutations shown are pArg121Trp, p.Val194Asp, p.Ile192Asn, pGlu134Lys, p.Ala219Asp, and p.Thr120Met. Proteins were detected with an anti-c-myc antibody, and the A-domain is indicated with an arrowhead.

To determine the effect of MED mutations on the secretion of matrilin-3 we cloned and expressed in mammalian cells full-length wt matrilin-3 and matrilin-3 harboring a MED mutation (p.Val194Asp) and non-synonymous polymorphism (p.Glu252Lys). SDS-PAGE and Western blot analysis of proteins from the cell media showed the presence of wt and p.Glu252Lys proteins at the expected molecular weight of ∼51 kDa, but not the p.Val194Asp protein (Fig. 1A). In contrast SDS-PAGE and Western blot analysis of proteins isolated from the cell lysate showed the presence of matrilin-3 containing the p.Val194Asp mutation (Fig. 1B), but not the wt and p.Glu252Lys proteins (other than at normal background levels expected for a cell expressing high levels of a recombinant protein).

These data therefore suggested that the p.Val194Asp mutation prevented the proper trafficking and secretion of matrilin-3, whereas as the p.Glu252Lys polymorphism did not.

### MED Mutations Disrupt the Folding of the Matrilin-3 A-Domain

The apparent clustering of the majority of MED mutations in the β-sheet led us to hypothesize that these substitutions were disrupting the folding of the A-domain of matrilin-3 (Table 1). Therefore, to determine the precise effect of these disease-causing mutations we expressed both normal and mutant A-domain proteins. We chose to express a number of MED mutations (p.Arg121Trp, p.Val194Asp, p.Thr120Met, p.Glu134Lys, p.Ile192Asn and p.Ala219Asp) in addition to the non-synonymous polymorphism (p.Glu252Lys).

Following SDS-PAGE and Western blot analysis of conditioned media from cell lines stably transfected with the wt A-domain construct a protein band was detected at approximately 25 kDa (Fig. 1C). This agrees with the predicted molecular weight of the A-domain (24.2 kDa including c-myc and His_6_ tags) and no multimers were observed in samples analyzed without DTT (data not shown).

SDS-PAGE and Western blot analysis of conditioned media and cell lysates from clones transfected with constructs containing the p.Arg121Trp, p.Val194Asp, p.Thr120Met, p.Glu134Lys, p.Ile192Asn and p.Ala219Asp mutations revealed that conditioned media contained no detectable A-domain (Fig. 1C). Instead the recombinant A-domain was detected in the cell lysates, suggesting that the mutant A-domains were not secreted but retained within the cell. In contrast, in those cells transfected with an expression vector containing the non-synonymous polymorphism (p.Glu252Lys), A-domain protein was detected in conditioned media (Fig. 1C), suggesting that the A-domain was secreted normally when it contained this non-disease-causing polymorphism. These data were therefore in agreement with those obtained for full-length matrilin-3 constructs.

### Retained Mutant A-Domains Are Primarily Unfolded

**Figure 2:**
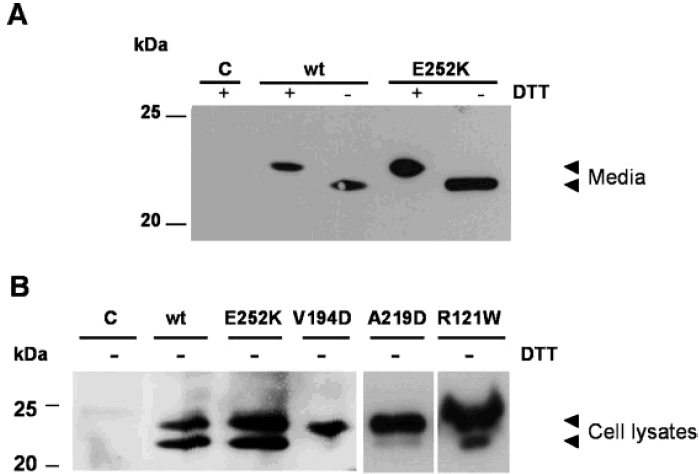
SDS-PAGE and Western blot analysis of conditionedmedia (**A**) and cell lysates (**B**) from CHO cells stably transfected with A-domain constructs under reducing (+) and non-reducing (−) conditions. Samples from cells expressing a normal A-domain construct (wt), a polymorphic construct (p.Glu252Lys), and constructs with the p.Val194Asp, p.Ala219Asp, and p.Arg121Trp mutations are shown. Samples from untransfected cells were used as a control (C).The recombinant protein was detected using an anti-c-myc antibody, andmolecular weights are shown in kDa.

SDS-PAGE and Western blot analysis of the wt A-domain showed that it migrated faster under non-reducing conditions, suggesting that the A-domain is smaller and more compact when the disulfide bond is intact (compare lanes 2 and 3 in Fig. 2A). This quantifiable characteristic of the recombinant A-domain therefore allowed us to study the disulfide bonded (“folded”) state of both the normal and mutant A-domains in greater detail. In the conditioned media, all of the wt A-domain, and also A-domain containing the non-synonymous polymorphism (p.Glu252Lys) ran as a faster migrating, disulfide bonded protein under non-reducing conditions (Lanes marked “–” in Fig. 2A). However, SDS-PAGE and Western blot analysis of lysates from cells transfected with wt or p.Glu252Lys A-domain revealed that non-reduced samples contained a mixture of both forms of A-domain in approximately equal quantities (Fig. 2B; lanes 2 and 3), which is characteristic of a cell expressing high levels of recombinant protein that is actively being folded and secreted.

On the other hand, when cell lysates from cells transfected with the mutant A-domains (p.Val194Asp, p.Ala219Asp, p.Arg121Trp) were analyzed by SDS-PAGE and Western blotting, the distribution of folded and unfolded A-domain was different to that of the wt A-domain (Fig. 2B; lanes 4–6). Mutant A-domains analyzed under non-reducing conditions appeared principally as the non-disulfide bonded and extended form (∼90%), with only a small proportion of the sample in the more compact form with the disulfide bond intact (∼10%), suggesting that the folding is either delayed or incomplete due to these mutations. In addition, disulfide bonded dimers of around 50 kDa and small quantities of trimers of around 75 kDa could also be observed (data not shown). These aggregates were not observed with either the wt or p.Glu252Lys A-domain proteins.

### Retained Mutant A-Domains Associate With the Chaperone ERp72

Since mutant A-domain was retained within the cell lysate and appeared incorrectly folded (i.e., probably without an intrachain disulfide bond); we examined the role that specific chaperone proteins may play in this process. Co-immunoprecipitation with various anti-chaperone protein antibodies was therefore used to identify putative interactions with the wt, polymorphic (p.Glu252Lys) and mutant A-domains (p.Val194Asp, p.Arg121Trp and p.Ala219Asp).

**Figure 3:**
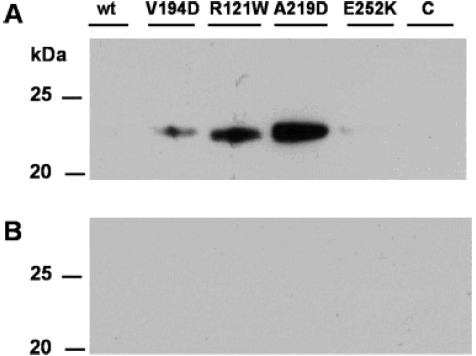
**A:** ERp72 was immunoprecipitated from the cell lysates of recombinant cells, and the immunoprecipitates were analyzed byWestern blot.The c-myc antibody detected recombinant A-domain in the R121W and A219D immunoprecipitates and to a lesser extent in theV194D immunoprecipitate. No band was detectable in the normal (wt), polymorphic (E252K), or control (C) lanes. **B:** Immunoprecipitation in the absence of anti-ERp72 antibody was performed as a control for nonspecific binding to theProteinA-agarose beads.Untransfected CHO cells were used as the control (C).

SDS-PAGE and Western blot analysis of total cell extracts revealed equivalent amounts of ERp72, PDI, Grp78 and Grp94 in all five cell lines (data not shown). However co-immunoprecipitation of the cell lysates with the anti-ERp72 antibody followed by SDS-PAGE and Western blot analysis of the immunoprecipitates showed that only the mutant A-domains co-precipitated with ERp72, whereas the wt or p.Glu252Lys A-domain did not (Fig. 3A). Immunoprecipitation experiments in the absence of the anti-ERp72 antibody demonstrated that the signal was not caused by non-specific binding of the A-domains to the agarose beads (Fig. 3B). Coimmunoprecipitation with antibodies to other chaperone proteins did not identify any specific interactions with mutant A-domains (data not shown).

### Mutant Matrilin-3 is Retained in the Rough Endoplasmic Reticulum InVivo

**Figure 4:**
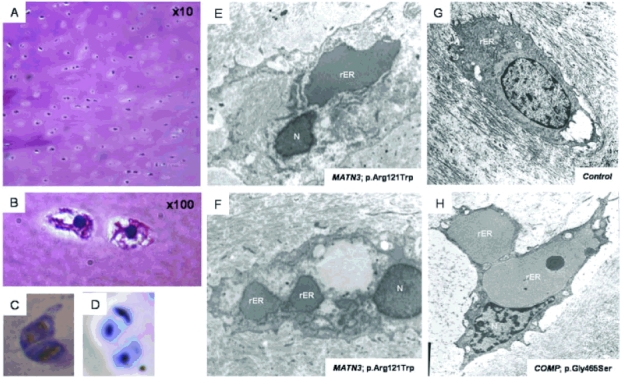
Light and electron micrographs of cartilage from the iliac crest biopsy of a 14-year-old patient with the p.Arg121Trp mutation. **A:** H&E staining of hyaline cartilage showing a normal matrix at × 10 magnification. **B:** Magnification (× 100) of the same section of cartilage showing a chondrocyte with apparent staining of the cytoplasm suggesting the presence of protein material, which is distinct from the nucleus (dark blue). **C:** Immunohistochemistry of the cartilage showing that the protein retained in the patient's chondrocyte stains with an antibody to matrilin-3 (brown), whereas there appears to be no intracellular staining for matrilin- 3 in the chondrocyte of an aged matched control sample (**D**) (the cell nucleus is stained blue by DAPI). **E** and **F:** Electron micrographs of the patients chondrocytes showing that the rough endoplasmic reticulum cisternae are swollen with accumulated protein that has a granular appearance, when compared to a control chondrocyte (**G**). For comparison an electron micrograph of a chondrocyte from a pseudoachondroplasia patient with a *COMP* mutation (p.Gly465Ser) is also shown and in this case the enlarged rER completely occupies the cytoplasm (**H**). rER: rough endoplasmic reticulum; N: nucleus. [Color figure can be viewed in the online issue,which is available at http://www.interscience.wiley.com.]

Hyaline cartilage from an iliac crest biopsy of a patient with the p.Arg121Trp mutation was analyzed using light and electron microscopy. In light micrographs the cartilage as a whole appeared normal following standard hematoxylin and eosin (H&E) staining (Fig. 4A). The cells have a normal chondrocyte morphology (i.e. rounded) and are distributed evenly throughout the extracellular matrix, which stains homogeneously [Morris et al., 2002]. However, at higher magnification the cytoplasm of the chondrocytes showed putative inclusions, which appeared to contain proteinaceous material (Fig. 4B). Immunohistochemical staining of this cartilage using an anti-matrilin-3 antibody confirmed that the retained material was matrilin-3 (Fig. 4C), which did not appear in the cytoplasm of chondrocytes of an age matched control (Fig. 4D). The resolution of these images was not sufficient to pinpoint the intracellular location of matrilin-3.

On electron micrographs, the inclusions noted at the light microscopy level appeared to be dilated cisternae of rough endoplasmic reticulum that contained flocculent electron dense material (Fig. 4E and F), which were not present in control chondrocytes (Fig. 4G). In addition there were numerous phagolysosomes within the chondrocytes but the Golgi was within normal limits. For comparison a chondrocyte from a pseudoachondroplasia patient with a COMP mutation is also shown (Fig. 4H). In summary therefore the chondrocytes appeared to have a normal morphology but with cytoplasmic inclusions that contained matrilin-3. Overall these in vivo findings confirm the in vitro expression data and suggest that misfolding of the A-domain results in the retention of matrilin-3 within the rER of chondrocytes.

## DISCUSSION

The multiple epiphyseal dysplasias are a relatively common and genetically heterogeneous group of inherited bone diseases and to date disease-causing mutations have been identified in six different genes, the products of which are important for cartilage structure and function [Briggs and Chapman, 2002; Unger and Hecht, 2001]. Autosomal dominant MED can result from mutations in the genes encoding cartilage oligomeric matrix protein (*COMP*), type IX collagen (*COL9A1, COL9A2* and *COL9A3*) and most recently matrilin-3 (*MATN3*) [Briggs and Chapman, 2002; Unger and Hecht, 2001]. In addition, an autosomal-recessive form of MED can result from mutations in the sulfate transporter 26A2 (*SLC26A2/DTDST*) [Ballhausen et al., 2003; Superti-Furga et al., 1999]. Although considerable progress has been made in understanding the disease mechanisms of MED caused by mutations in *COMP* [Unger and Hecht, 2001] and *SLC26A2* [Forlino et al., 2005; Rossi and Superti-Furga, 2001], virtually nothing is known about the disease mechanisms of MED caused by mutations in *MATN3*. The aim of this study therefore was to determine the molecular cell pathology of MED caused by *MATN3* mutations.

All of the MED mutations in *MATN3* are missense mutations found in exon 2, which encodes the single A-domain of matrilin-3 [Chapman et al., 2001; Jackson et al., 2004; Mabuchi et al., 2004; Mostert et al., 2003]. To study the effect of these mutations on the secretion of matrilin-3 we cloned and expressed full-length wt, mutant (p.Val194Asp) and polymorphic (p.Glu252Lys) matrilin-3 in mammalian cells. Both the wt and p.Glu252Lys proteins were efficiently secreted, whereas the p.Val194Asp protein was retained in the cell lysate, suggesting that the mutation disrupts the secretion of the abnormal protein. These data are similar to those obtained for *COMP* mutations [Hecht et al., 2005] and suggest a common disease mechanism for this bone dysplasia family.

Most of the MED mutations in *MATN3* affect conserved residues in one of the six β-strands (βA-βF) that together comprise the β-sheet of the A-domain [Jackson et al., 2004]. This precise grouping of MED mutations in the β-sheet led to us to hypothesize that they might disrupt the folding of this structurally important domain. To investigate this observation further we expressed individual A-domains; either wt or containing a disease-causing mutation (p.Thr120Met, p.Arg121Trp, p.Glu134Lys, p.Ile192Asn, p.Val194Asp and p.Ala219Asp) or non-synonymous polymorphism (p.Glu252Lys). Normal A-domain was expressed and secreted from mammalian cells as a disulfide bonded monomer, demonstrating that this domain can fold independently of the other matrilin-3 modules (i.e., EGF-like motifs and coiled-coil domain). However, in all cells expressing a mutation the recombinant A-domain was retained within the cell lysate and no protein was detected in the cell culture media. These data are therefore in agreement with those obtained for full-length protein and confirm that *MATN3* mutations disrupt the secretion of abnormal matrilin-3 by causing misfolding of the A-domain.

Interestingly, SDS-PAGE of cell lysates under nonreducing conditions identified both a disulfide bonded (“folded”) A-domain and a non-disulfide bonded (“unfolded”) intermediate. This measurable characteristic of the expressed A-domains provided us with the means to study further the effect of the specific disease-causing mutations on the folding of single A-domains. Analysis of the retained mutant A-domains demonstrated that they existed primarily as non-disulfide bonded folding intermediates (approximately 9/1: nondisulfide/disulfide bonded). In addition disulfide bonded dimers and trimers were also detected, suggesting a potential mechanism for the aggregation of misfolded matrilin-3 within the rER.

In contrast, recombinant A-domain containing p.Glu252Lys, a non-disease-causing polymorphism, was secreted in a similar manner to normal A-domain and also had a similar proportion of non-disulfide bonded protein in the cell lysate. This polymorphism results in an amino acid substitution in one of the external α-helices of the A-domain (α-7) and we hypothesize that such a substitution is unlikely to prevent the correct folding of the A-domain. Interestingly a recent study has reported a MED-causing mutation (p.Phe105Ser) in the α1 helix of the matrilin-3 A-domain in one Japanese family [Mabuchi et al., 2004]. This mutation affects a highly conserved phenylalanine residue, which unlike Glu252 may be important for the structure and/or function of matrilin-3. Structural and functional analysis of p.Phe105Ser will help elucidate the role of this mutation in the pathogenesis of MED.

Since mutant A-domains were persisting as non-disulfide bonded (“unfolded”) intermediates we decided to examine the role that specific chaperone proteins may play in this process [Kuznetsov et al., 1997]. In particular we were interested in chaperone proteins that are specifically involved in catalyzing the formation of disulfide bonds [Clissold and Bicknell, 2003; Ferrari and Soling, 1999]. Analysis by coimmunoprecipitation revealed that only mutant A-domain formed a stable complex with ERp72. ERp72 is an abundant endoplasmic reticulum protein that contains three copies of the active site sequences of protein disulfide isomerase and plays an important role in catalyzing the formation of disulfide bonds [Mazzarella et al., 1990]. In addition it has also been shown to play a key role in the unfolded protein response (UPR) and has in fact been implicated in the retention of misfolded COMP in the chondrocytes of pseudoachondroplasia (PSACH) and MED patients resulting from mutations in the type III repeats of COMP [Hecht et al., 2001]. Our data therefore suggest that mutant A-domains experience a disruption in folding, which hinders the formation of intrachain disulfide bonds but still allows the formation of inappropriate interchain disulfide bonds between other A-domains thus producing both dimers and trimers. However we were not able to determine whether binding of ERp72 to mutant A-domain is a cause or a consequence of the delayed folding.

Although our initial studies were focused on understanding the disease mechanism *in vitro*, the chance to study cartilage from an MED patient with a *MATN3* mutation (p.Arg121Trp) provided us with the opportunity to confirm these findings in vivo. Analysis by light and electron microscopy revealed the retention of protein within the rER of chondrocytes. Furthermore immunohistochemistry revealed that the retained protein stained with antibodies against matrilin-3. These data therefore confirm our in vitro findings and suggest that *MATN3* mutations result in the misfolding of the matrilin-3 A-domain, which subsequently results in the retention of matrilin-3 oligomers in the rER of patient chondrocytes.

Therefore, the molecular cell pathology of MED caused by *MATN3* mutations has similarities to skeletal dysplasias caused by mutations in the genes encoding other extracellular matrix structural proteins, particularly PSACH and forms of MED that result from mutations in COMP [Unger and Hecht, 2001]. In these allelic diseases, mutations in the type III repeats of COMP result in the persistence of an unstructured Ca^2+^ binding domain [Kleerekoper et al., 2002]. These abnormal COMP molecules are then retained within the rER of cells via the unfolded protein response [Dinser et al., 2002]. The primary accumulation of abnormal COMP also appears to result in the secondary retention of type IX collagen and other proteins, eventually producing large insoluble “inclusion bodies” [Vranka et al., 2001]. More recently, Hecht et al. [2005] showed that mutant COMP can also dramatically affect the secretion of normal matrilin-3 from PSACH chondrocytes. This mass accumulation of protein eventually results in cell death and a reduction in the number of viable cells in the growth plate [Duke et al., 2003]. Interestingly a number of chaperone proteins have been shown to be involved in this process including ERp72 [Hecht et al., 2001; Vranka et al., 2001].

MED is perhaps the most genetically heterogeneous of all autosomal dominant skeletal dysplasia phenotypes and missense mutations have now been identified in the genes encoding matrilin-3, COMP and type IX collagen [Briggs and Chapman, 2002; Unger and Hecht, 2001]. Recently there has been much speculation about the reason for this genetic heterogeneity, and a number of hypotheses have been suggested; i.e., 1) some mutations disrupt specific interactions between matrilin-3, type IX collagen, and COMP in the cartilage ECM [Briggs and Chapman, 2002]; 2) the blocked/delayed secretion of one mutant protein results in a cascade of protein retention within the rER of chondrocytes [Unger and Hecht, 2001]; or 3) a combination of both mechanisms [Dinser et al., 2002]. In summary, our data suggest that the retention of mutant cartilage structural protein, within the rough endoplasmic reticulum, is a common disease mechanism that is shared between MED resulting from either COMP or *MATN3* mutations.
